# Combination of conservative and surgical methods in the treatment of giant lymphedema of the scrotum: A case report

**DOI:** 10.3389/fsurg.2023.1048159

**Published:** 2023-04-12

**Authors:** Andrey L. Istranov, Ivan G. Makarov, Natalya V. Makarova, Inna Tulina, Ilya V. Ulasov, Yuliya I. Isakova

**Affiliations:** ^1^ Department of Oncology, Radiotherapy and Reconstructive Surgery, Sechenov First Moscow State Medical University, Moscow, Russia; ^2^ Clinical-Research Center for Rehabilitation of Lymphedema Patients “LYMPHA”, Moscow, Russia; ^3^ Clinic of Colorectal and Minimally Invasive Surgery, Department of Surgery, Sechenov First Moscow State Medical University, Moscow, Russia; ^4^ Group of Experimental Biotherapy and Diagnostic, Institute for Regenerative Medicine, Sechenov First Moscow State Medical University, Moscow, Russia; ^5^ World-Class Research Center “Digital Biodesign and Personalized Healthcare”, Sechenov First Moscow State Medical University, Moscow, Russia

**Keywords:** genital lymphedema, reconstructive surgery, penis, scrotum, phalloplasty, scrotoplasty

## Abstract

**Objective:**

Genital lymphedema is a severe, disabling condition associated with a malfunction of the lymphatic system. Primary lymphedema of the scrotum is a variant of congenital dysplasia of lymphatic vessels. Secondary genital lymphedema is much more common and can be caused by parasitic invasion (filariasis) or damage to the lymphatic system during the treatment of cancer (radiation therapy, lymphadenectomy). Healthcare providers are frequently unable to detect and treat this illness successfully in ordinary clinical practice. This paper uses the case of a patient with stage 3 secondary lymphedema (unknown genesis) of both lower extremities and lymphedema of the scrotum, complicated by recurrent erysipelas, a history of lymphorrhoea, impaired skin trophic and multiple papillomatosis, to demonstrate the efficacy of a combination of conservative and surgical methods in the treatment of giant lymphedema of the scrotum.

**Methods:**

In the treatment, the combination of decongestant physical therapy (CDPT, CDT) according to M. Földi was used at pre-surgery and post-surgery stages, combined with a reconstructive operation, including the removal of the affected tissues of the urogenital region, phalloplasty, and scrotoplasty with rotational skin flaps.

**Results:**

A decrease in the circumference of the lowest extremities in the lower leg area by 68 cm on the right and by 69 cm on the left was achieved by conservative treatment. Due to the combination of conservative and surgical treatment, the patient's body weight decreased by 69.4 kg, and the scrotum decreased by 63 cm. Subsequently, the patient fully recovered his sexual function.

**Conclusion:**

A combination of complex decongestive physical therapy and surgery is necessary for patients with advanced genital edema. The isolated use of surgical or conservative treatment does not provide a sufficient improvement in the patient's quality of life. Modern plastic surgery technologies enable patients to achieve complete functional and cosmetic recovery, while proper selection and usage of compression hosiery help preserve and improve the outcomes acquired following treatment.

## Introduction

Genital lymphedema is a severe, disabling condition associated with a malfunction of the lymphatic system. Primary lymphedema of the scrotum is a variant of congenital dysplasia of lymphatic vessels, which leads to impaired lymph circulation and can be combined with lymphedema of the lower extremities (1, 2). Secondary genital lymphedema is much more common and can be caused by parasitic invasion (filariasis) or damage to the lymphatic system during the treatment of cancer (radiation therapy, lymphadenectomy). With the progression of edema, the skin of the scrotum and penis thicken, loses its elasticity, and becomes susceptible to various infections because of an impaired local immune response. With a significant increase in the volume of the scrotum, the penis is drawn into the surrounding edematous tissues. Such changes lead to a decrease in the patient's quality of life due to limited mobility, problems with urination, the inability to have sexual intercourse and social maladjustment (3). This condition negatively affects not only the patient's physiology but also his psychological condition (and may even cause depression), infectious complications, such as erysipelas and lymphangitis. Inflammatory processes occurring in the skin and subcutaneous adipose tissue lead to loss of tissue elasticity, hyperplasia of collagen connective tissue, and the development of fibrosis, which cause irreversible changes and progressive loss of function of the affected tissues (4).

The treatment of genital lymphedema can be divided into two major groups: conservative and surgical. In the overwhelming majority of cases, the conservative treatment uses the options of physiotherapeutic therapy based on equipment available in the clinic (laser and magnetic therapy, intermittent pneumocompression), massage, therapeutic exercises, reflexology, and drug therapy. As a rule, conservative therapy allows for significant improvement in the patient's condition, especially in the early stages of the disease. An important aspect is an integrated approach in the choice of conservative techniques (5) We use complex physical decongestive therapy (Complex Decongestive Therapy according to M. Földi), which allows not only to achieve a reduction in edema by more than 75%, but also to maintain the results achieved during the treatment.

The surgical management of lymphedema is classified as either physiologic (reconstructive) surgery aimed at restoring lymphatic drainage—lymph node transfer or lymphaticovenous bypass/anastomosis or reductive (excisional or ablative) surgery aimed at a resection of the altered tissues and reconstruction of the scrotum with a goal of restoring the appearance of the genitals (6). This operation is widely used in the treatment of scrotal lymphedema; however, the main problem with the isolated use of surgical methods for the treatment of lymphedema is the high risk of postoperative complications and further progression of lymphedema.

## Case description

**Figure 1:**
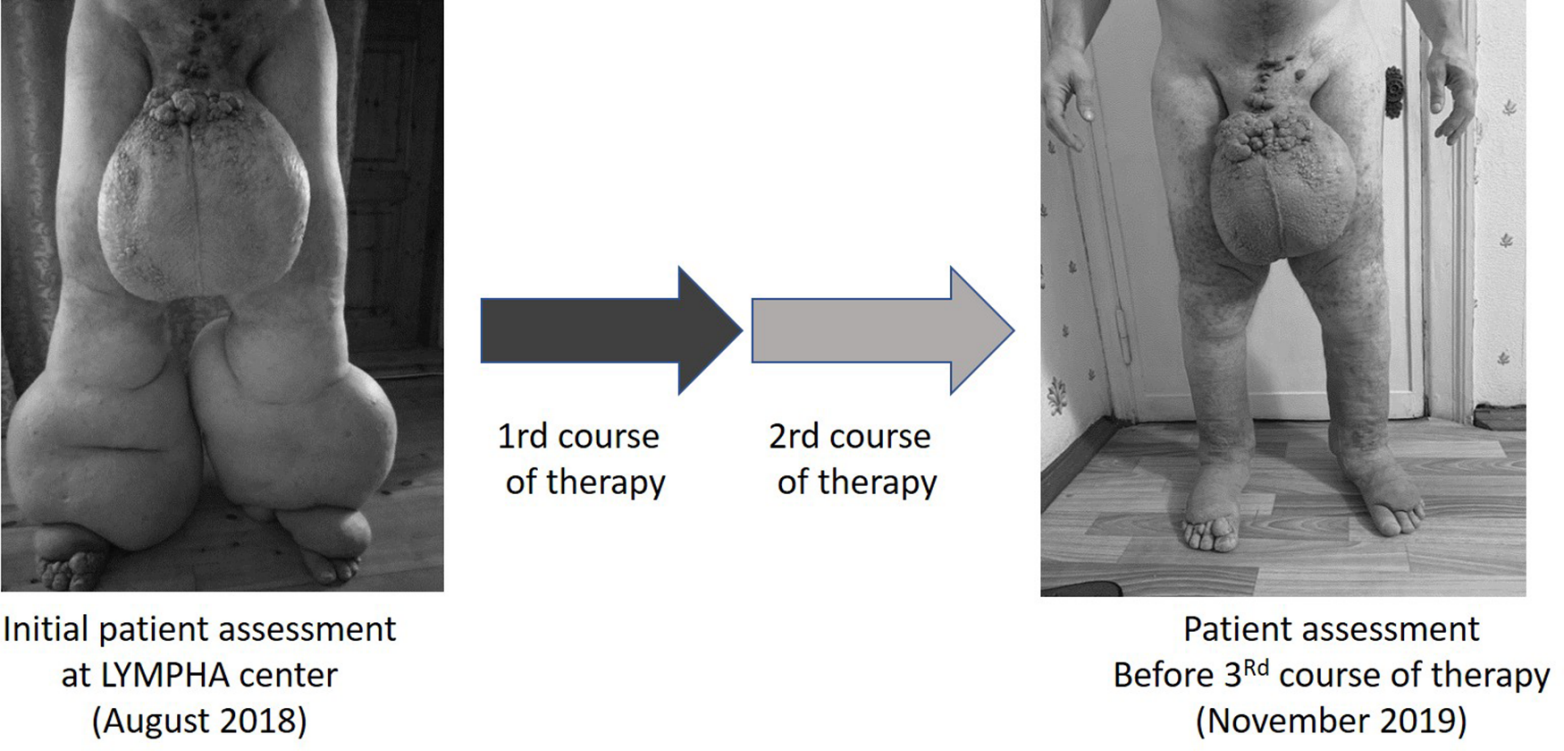
Patient's conditions before and at the time of treatments. Microphotography patient assessment at the time of hospital admission (**A**, August 2018) and before 3rd course of therapy (**B**, November 2019).

The 48-year-old male patient (Figure 1), first sought medical advice at the Research and Clinical Center LYMPHA in 2018. It is known from his past medical history that in 1989, when he was 17 years old. At that time, the patient developed a fist-sized mass on the right side of the groin area after swimming in the sea. The patient underwent a bilateral biopsy of inguinal lymph nodes, after which he developed swelling in the thighs on both sides. Subsequently, the edema gradually spread to his legs and feet. The patient consulted specialists at the Vishnevsky Research Institute, where elastic bandaging of the lower extremities was recommended. The patient followed the recommendations accurately. In 2010, the edema progressed to the genital area. The patient suffered repeated episodes of erysipelas and transient lymphorrhea. In 2017, he turned to a family planning center for an *in vitro* fertilization (IVF) procedure, and after surgery, the edema in the genitals and lower extremities increased significantly. Based on our knowledge, there are no inherent risks possessed for the patient safety using surgical procedures.

## Surgical methods

**Figure 2:**
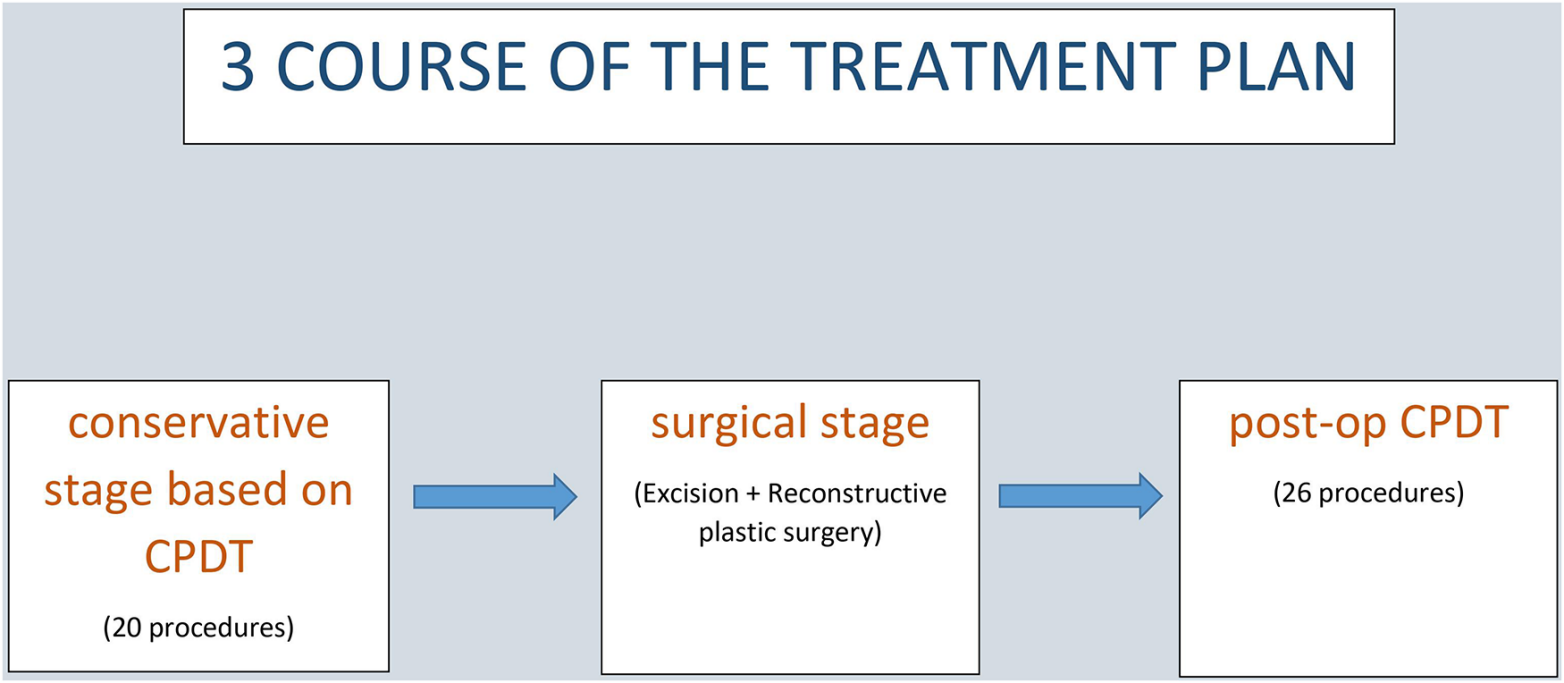
Flow diagram illustrating the multiphase therapeutic methods approach to the development of treatment plan against secondary genital lymphedema. CDPT, combination of decongestant physical therapy.

In the treatment, the combination of decongestant physical therapy (CDPT, CDT) according to M. Földi was used at pre-surgery and post-surgery stages (Figure 2), combined with a reconstructive operation, including the removal of the affected tissues of the urogenital region, phalloplasty, and scrotoplasty with rotation skin flaps. The rotation flap of the inguinal region was used for scrotum reconstruction. During surgery treatment, the all altered integumentary tissues were removed, including the skin, subcutaneous fat, and scrotum membranes. Cavernous bodies and urethra, as well as testicles with spermatic cord, were left.

## Timeline

Figure 2.

## Diagnostic/therapeutic assessment

### First course of treatment

**Figure 3:**
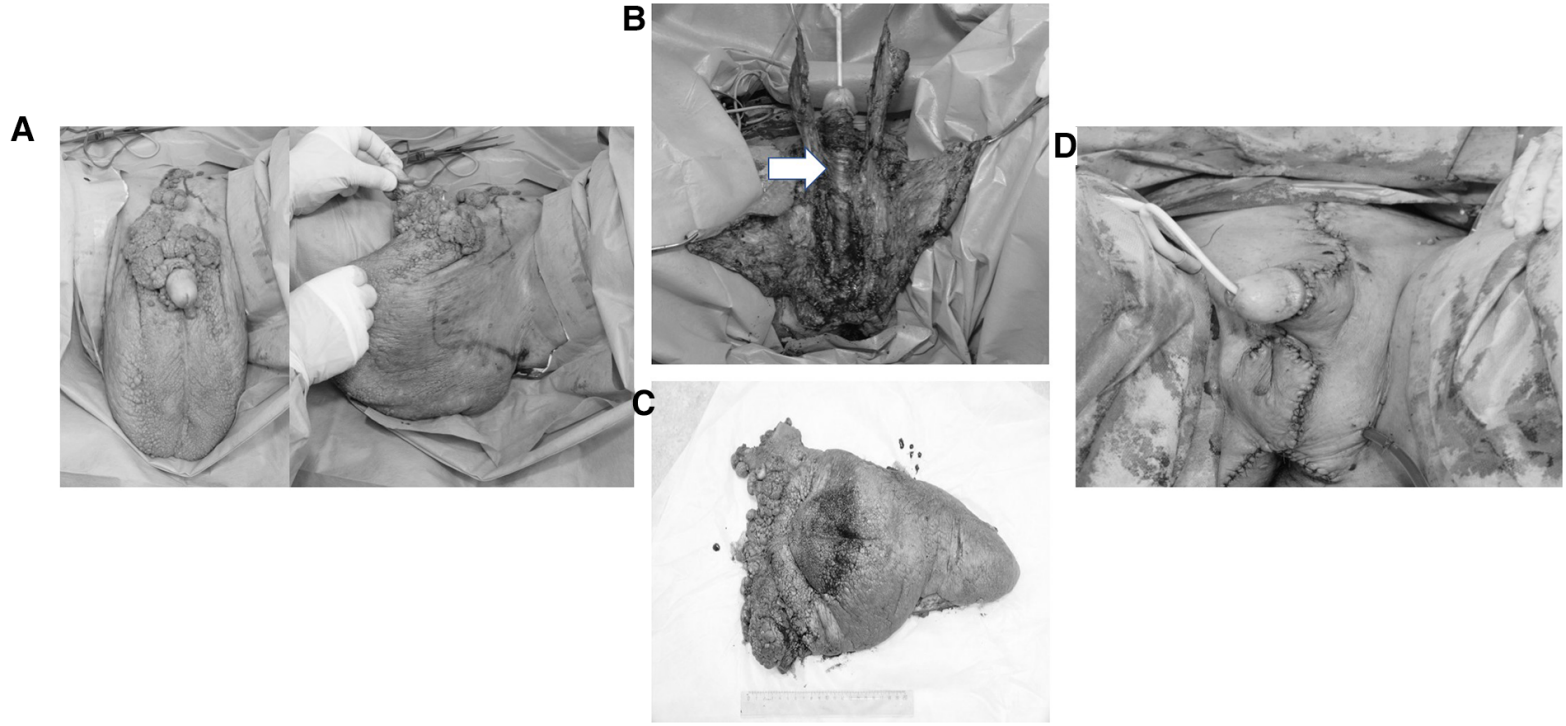
Dynamics of tumor resection over a course of surgery. Intraoperative photos were taken to select the resection line (**A**), to demonstrate the excess of soft tissue (**B**), removed soft tissue fragment (**C**) and the outcome of resection (**D**). Naked structures of the penis and dissected testicles are selected by arrow.

At the time of admission in August 2018, the patient's skin was dark-colored and dry with multiple papillomatoses of the lower extremities and scrotum. The patient had severe, dense edema of the lower extremities, deforming their anatomical contour; elephantiasis, and severe edema of the scrotum. The body temperature was not changed, the sensitivity of the skin was reduced in the edematous area. The contour of the ankle and knee joints was smoothed, the skin folds hung over the ankle and knee joints, the Stemmer symptom was positive on both sides, the regional lymph nodes were not palpable. On October 11, 2018, we detected a 4 × 4 cm ulcerative defect on the front surface of the right shin, and a biopsy was collected. Further analysis did not confirm the development of lymphangiosarcoma. The lower extremities and scrotum had multiple fibrosed lymphocytes. A total of 30 complex physical decongestive procedures were performed, including manual lymphatic drainage, compression bandaging, and skin care. A positive response to treatment was noted with softening of the tissues and a significant decrease of the edema's circumference in the left lower limb: from 8.5 cm to 54.0 cm, and in the right lower limb: from 11.0 cm to 52.5 cm. The patient's body weight decreased by 51.7 kg. After the course of treatment, flat-knit compression hosiery Mediven 550 made to individual measure was put on, including the 2nd class of compression pants, the 4th class of compression knee-high stockings, and the 3rd class of compression stockings, on both lower extremities (Figure 3).

### Second course of treatment

**Figure 4:**
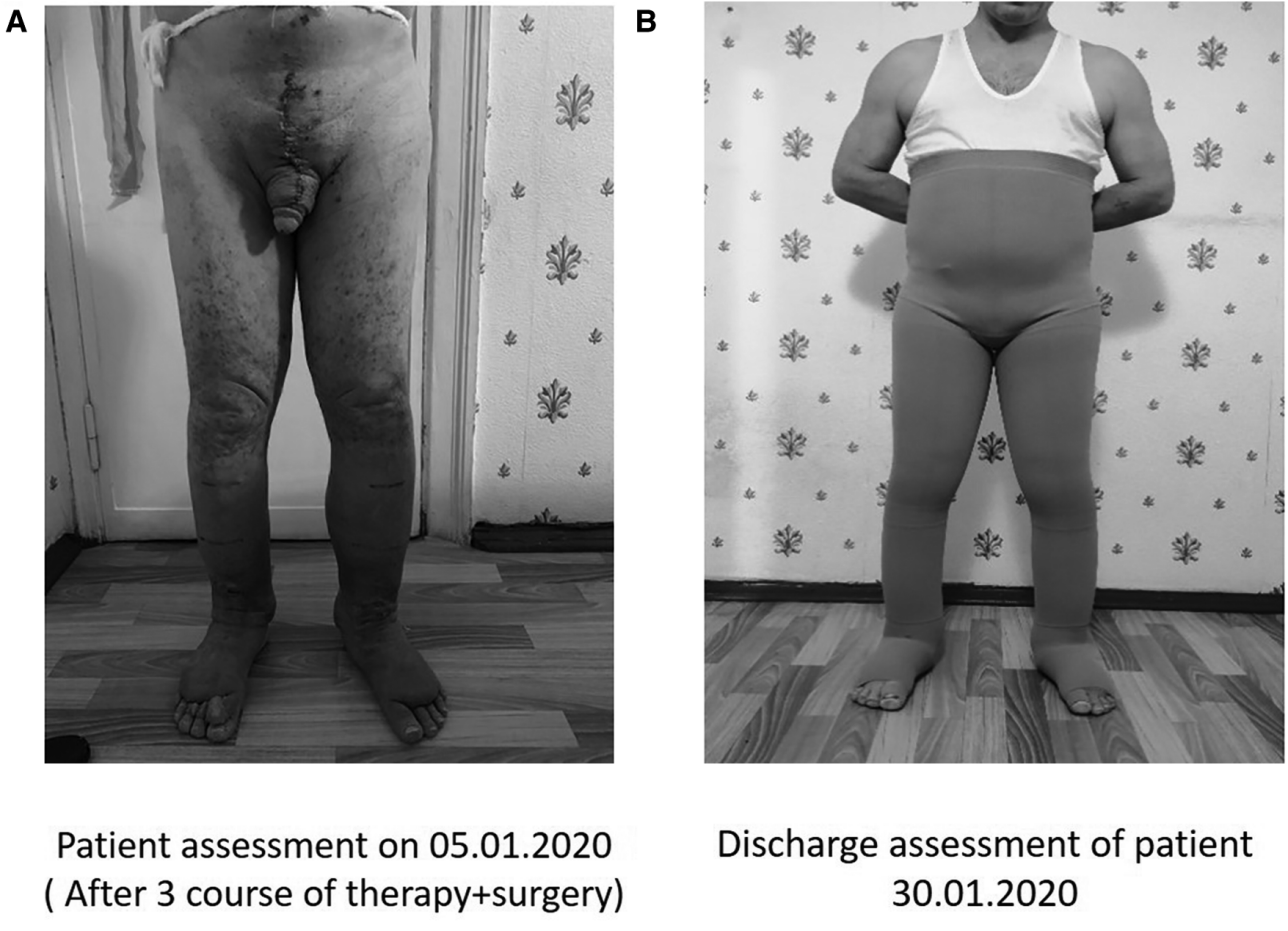
The patient's conditions a month after surgery (January 05, 2020, **A**) and at the time of discharge (January 30, 2020, **B**). Patient wearing the compression knitwear flat knitting Mediven 550, made according to individual standards—leggings of 2 compression class, socks of 3 compression class, product on the foot of 3 compression class on the left lower limb, stockings of 3 compression classes.

The second course of treatment was performed six months later, in January 2019. A positive response to treatment was noted with softening of the soft tissues and a significant decrease in edema on the left lower limb (from 1.5 cm to 18 cm) and the right lower limb (from 3.0 cm to 17 cm). The circumference of the scrotum decreased by 2.5 cm. The patient's body weight decreased by 9.7 kg. After the course of treatment, flat-knit compression hosiery Mediven 550 made to individual measure was put on, including the 3rd class of compression stockings, the 4th class of compression knee-high stockings, the 2nd class of compression CT hosiery (mid-leg leggings), and the 2nd class of compression sock on the left foot (Figure 4, Table 1).

### Third course of treatment

In November 2019, the patient's treatment included a conservative stage based on CPDT and a surgical stage involving the excision of excess soft tissues and reconstructive plastic surgery of the scrotum and penis. At the time of admission, the patient's edema was increased (compared with his February 2019′s medical examination findings, up to +9.5 cm on the right lower limb, and up to +6 cm on the left lower limb with the scrotum volume increased by 30 cm).

The patient was admitted to the Research and Clinical Center “Lympha” for the third course of treatment on November 5, 2019. Overall, around 20 procedures of complex physical decongestive therapy (manual lymphatic drainage, compression bandaging, and skin care) were performed, as a result of which the volume of the lower limbs and scrotum decreased by 10.5 cm on the right lower limb, by 6.0 cm on the left lower limb, and the scrotum volume decreased by 29 cm.

In December 2019, the patient was admitted to the Department of Plastic and Reconstructive Surgery of University Clinical Hospital No. 1 of the Federal State Institution of Higher Education I.M. Sechenov University of the Ministry of Health of Russia (Sechenov University) for planned surgical treatment. The patient underwent a routine pre-surgery examination, during which no contraindications to surgery were found. On December 5, 2019, reconstructive surgery was performed, including the removal of the affected tissues of the urogenital area, phalloplasty, and scrotoplasty (plastic surgery of the penis and scrotum). During the surgery, incisions were made in the pubic area, in the penis up to the glans, and in the area of the scrotum, according to preliminary markings. Excess soft tissues were removed. The anatomical structures of the penis were exposed; the testicles were dissected. In healthy soft tissues of the inguinal folds, penis, skin of the scrotum, and pubic area that were not included in the resection zone, multiple neoplasms were excised. All visible lymphatic vessels were carefully coagulated. The scrotum was formed with local tissues and rotation flaps, and the testicles were placed in the formed pocket. The deficiency of the cover tissues of the penis was supplied by the rotation of skin flaps in the groin area. No additional skin grafts were required. A Foley urinary catheter No. 14 was installed. A thorough hemostasis was performed, followed by layer-by-layer tissue suturing. The removed excess soft tissue and individual removed neoplasms were sent for histological examination.

In the post-surgery period, the patient received antibacterial and analgesic therapy. For the regenerative processes, the stimulating agents such as solcoseryl or actovegin gels were used. Daily dressings of the post-surgery wound were performed using aseptic solutions and wound-healing ointments.

The results of a pathomorphological study of surgically removed soft tissues performed on December 9, 2019, showed skin fragments with papillomatous growths of the epidermis, keratinization, acanthosis, and signs of viral lesions. In the dermis, fibrosis with areas of myxomatosis, an abundance of dilated lymphatic vessels, and perivascular lymphoid infiltration were found. At the edge of the resection in the epidermis, there were signs of Grade 2 dysplasia. Taking into account the clinical findings, the morphologic changes are consistent with Buschke–Lovenstein giant condyloma and chronic lymphedema.

Later on, the patient underwent dressings, as well as manual lymphatic drainage, and compression bandaging (26 procedures). The wound healed by primary intention; the sutures were removed on the 14th day after the surgery. There were no complications in the postoperative period. New custom-made compression hosiery was put on. The hospital stay for the patient has lasted 3 days with outpatient follow-up and daily dressings for 2 weeks. The patient was observed for two weeks following the operation with subsequent follow-up examinations one, three, and six months later. During the treatment, which included both conservative and surgical stages, a decrease in the volume of the right lower limb by 10.5 cm, the left lower limb by 6.0 cm, and a decrease in the scrotum volume by 62 cm was achieved. The patient's weight was reduced by 21.2 kg. Erectile function was recovered 30 days after surgery. The patient was discharged in good health with instructions to wear compression hosiery at all times during the day and return in three months for an evaluation.

In just three courses of treatment, the last of which included a surgical stage, circumference of the lower limbs decreased by 68.5 cm in the right lower limb, by 69 cm in the left lower limb, and the scrotum decreased by 63 cm. The patient successfully conceived and gave birth to a daughter a year after the intensive therapy.

## Discussion

Lymphedema of the scrotum is a condition in which there is a progressive increase in the volume of the soft tissues of the scrotum and penis. This pathology can be congenital or acquired. The cause of the congenital form of the disease is usually associated with a hypoplasia of the lymphatic system. The acquired forms are caused by iatrogenic injuries as a result of tissue dissection, lymphadenectomy, or irradiation of this area during cancer progression. There are also acute and chronic forms of lymphedema. Often, this pathology is combined with lymphocytosis of the lower extremities.

In the field, the various options for correcting this lymphedema have been described and involved conservative therapy, reconstructive operations in the perineum (resection of excess soft tissues with tissue restoration using local tissues), the imposition of lymph-venous anastomoses, and lymph node transplantation.

The most common treatment for this disease is a surgical resection of scrotal and penile soft tissues. This method allows you to eliminate the thickened skin. At the same time, the testicles and body of the penis are isolated, then, using local tissues, a new scrotum can be formed. At this stage, it may be difficult to plan flaps to recreate the scrotum, since the affected local tissues do not heal well, and relapse may occur. Due to the specific thermoregulation ofit is impractical to use a skin flap from another anatomical area with bigger thickness and differential thermoregulation.

Some authors suggest techniques to eliminate the lack of integumentary tissues of the penis using free split skin grafts. In the presence of unchanged skin of the penis, it is possible to use *Z*-plastic to minimize the risk of contracture and retraction of the penis along the median line. These techniques have proven themselves well and allow us to achieve high results (2, 3). In our clinical observation at the surgical stage, we adhered to the same principles of reconstruction, but to close the defect of the penis, we used a rotation flap of the inguinal region, where the skin was not affected. This modification has several advantages over split skin graft. The rotation flap is vascularized, which provides better engraftment.

The mobility of superficial tissues is preserved. The size of the penis does not decrease due to the thickness of the flap.

To summarize the key elements of our protocol to highlight the strong and weak sides of our protocol:

Advantages of using the rotation flaps are:

• It contains a well revascularized tissues;
• The tissue donor site is located in the immediate vicinity of the defect, which will be eliminated. (to disregard to transplant free flaps and to avoid the appearance of new defects in remote areas of the body);
• Straight forward surgical procedure that does not require a special equipment;
• The size of the inguinal flaps allows you to eliminate defects of almost any size in the urogenital region;
• The thickness of the inguinal flaps is optimal for recreating the cover of the penis.

Limitations are:

• Current any pathological process in the skin of potential flaps
• There is a lack of adequate blood supply to the tissues of the inguinal region (vascular damage or their developmental abnormality).

Conservative therapy is considered an ineffective method of treating lymphedema of the scrotum. We preferred to combine conservative and surgical components. In agreement with our findings, physiotherapy therapy can result in a large reduction in scrotal volume, considerably simplifying the surgical stage of repair.

For the first time, lymph-venous anastomosis was used to treat genital lymphedema in men by Huang et al. They performed an anastomosis of superficial lymphatic vessels with small veins in the subcutaneous tissue (7). Mulenga et al. selected deep lymphatic vessels running along the spermatic cord and anastomosed them with the dowsing venous plexus, which also runs alongside the spermatic cord (8). According to studies of the lymphatic system of the inguinal region, the diameters of the superficial lymphatic vessels are smaller (from 0.2 to 0.5 mm) compared to the deep lymphatic vessels (from 0.5 to 1 mm). Thus, building a lymphovenous shunt is technically more reliable when using deep lymphatic vessels. However, lymphatic outflow can only be restored with lymphovenous anastomosis in instances where there is little stagnation, well-isolated lymphatic channels, and no fibrosis (9).

## Conclusion

Giant lymphedema of the scrotum is a serious condition that causes both physical and psychological discomfort, including the loss of erectile function and disability. After our comprehensive treatment, the patient noted a significant improvement in his quality of life. This case demonstrates the importance of the optimal combination of conservative and surgical treatment for giant scrotal lymphedema. Conservative treatment is necessary to minimize the edema, which makes it possible to perform the surgical stage more successfully, minimizing the risk of suture disruption and prolonged lymphorrhea. Correct compression hosiery selection and use allow not only for maintenance but also to improve the results obtained during treatment, whereas modern plastic surgery methods may provide patients with complete functional and aesthetic rehabilitation.

### Patient perspective

The patient described her perspective 3 months post-operatively. Her main messages are summarized here, in the order in which they were expressed, as we think they are valuable for clinicians:

The most annoying feeling was that no one believed me.”

1. “My doctors were reluctant to send me for imaging examinations, probably because they didn't believe that anything was wrong with me or maybe because of financial considerations.”
2. “It still amazes me that none of the imaging examinations revealed the strange body.”
3. “It was extremely important to me when, finally, one of the doctors looked at the imaging tests’ CDs and did not rely solely on the radiologist's printed interpretation.”
4. “It was exciting and touching when, finally, someone believed me after 8 years.”
5. “The suffering was immense. I couldn't engage in sports and work out or even play frisbee at the beach.”
6. “I feel much better and most of the time have no complaints. However, as strange as it is, and although I know that the foreign body is no longer there, I can sometimes still feel the pain, and even dream about the foreign body wandering in my breast.”
7. “This is a very traumatic event. I wonder if it wouldn't have been better to endure a worse problem or even an infection but to have been quickly diagnosed with a shorter torment time.”
8. After the treatment, I felt incredible relief and emotional uplift. I'm back to normal. I can wear normal clothes and move around freely.

**Table 1:** Dynamics of the size of the lower extremities during the treatment period.

Milestone/day of observation	Left lower limb/right lower limb								
	Foot, sm	Lower third of the lower leg	Middle part of the lower leg	Upper part of the lower leg	Lower third of the thigh	Middle third of thigh	Upper part of the thigh	Scrotum circle	Weight
Before treatment August 30, 2018	41/39	104/103	107/93	68/59	70/76.5	69/71.5	69.5/71	71	166.7
After treatment October 12, 2018	33/27.5	53.5/58	64/56.5	56/47.5	50/52	57/58	61.5/60	47	118
Before treatment January 21, 2019	28/28.5	38.5/43	42.5/43.5	44/43.2	43.2/64	67/68.5	68.5/68.5	60	121
After treatment February 15, 2019	26.5/25	34/36	40.5/40.5	41/41	47/47	58/58	62/62	43	110
Before treatment November 05, 2019	29.5/29.5	39/38.5	43/38	44.5/42	46/56.5	64/65	66.5/68.5	70	118.5
Preoperative after 20 КФПТ December 02, 2019	27.5/27.5	36.5/38	42/38	42/40	47/47	58/59.5	63/63.5	40	106
After surgery December 18, 2019	29/28	34/33	41/44	42.5/46	50.5/50	60.5/59	68/65	11	9,805
Preoperative after 26 КФПТ January 30, 2020	28.5/28.5	35/34.5	41/38	41.5/40	47/47	58/59	66/66	8	97.3
Dynamic over a course of therapy	−12.5/10.5	−69/−68.5	−66/−55	−23/−29.5	−23/−29.5	−11/−12.5	−3.5/−5	−63	−69.4

## Data Availability

The raw data supporting the conclusions of this article will be made available by the authors, without undue reservation.
